# The complete mitochondrial genome sequences of *Brassica napus* varieties NY18 and 088018

**DOI:** 10.1080/23802359.2021.1912672

**Published:** 2021-04-15

**Authors:** Xiaoying Zhou, Song Chen, Sanxiong Fu

**Affiliations:** Institute of Industrial Crops, Jiangsu Academy of Agricultural Sciences, Key Laboratory of Cotton and Rapeseed, Ministry of Agriculture and Rural Affairs, Nanjing, China

**Keywords:** *Brassica napus* variety NY18, variety 08818, complete mitochondrial genome, phylogeny Brassicaceae

## Abstract

*Brassica napus* variety NY18 and 088018 are female and male parents of the national registered variety Ningza 1818, respectively. Here, we determined the complete mitochondrial genomes of these two varieties. The genome sizes of NY18 and 088018 were 221,864 bp and 222,015 bp, respectively. Both genomes contained 40 protein-coding genes, 21 tRNA genes, and three rRNA genes. Considerable structural variations existed between the two mitochondrial genomes, which were separated into five syntenic regions. Phylogenetic analysis using the maximum-likelihood method showed that the mitochondrial sequences of *B. napus* were closely clustered, forming a single clade which had a relatively close relationship with the clade formed by *B. rapa*, *B. juncea*, and *B. oleracea*.

Oilseed rape (*Brassica napus* Linnaeus 1753. AACC, 2n = 38) is not only the second most important oilseed crop worldwide for human nutrition and industrial products, but also an emerging biofuel crop (Weiss 2000). In China, the cultivated area of oilseed rape reaches 67 million hectares, with an annual yield of around 4.5 million tons (Fan et al. 2018). The genetic diversity of oilseed rape underlying its massive phenotypic variations remains largely unexplored. But the mitochondrial genome-based phylogenetic analysis would improve our understanding of the evolutionary relationship of this plant and accelerate the genetic improvement of *B. napus. Brassica napus* variety NY18, selected from variety NY10, is a female parent of the national registered variety Ningza 1818. And 088018 selected from variety NY7 × Marnoo, is a male parent of the national registered variety Ningza 1818 (Fu et al. 2015). The characterization and comparison of the complete mitochondrial genomes of varieties NY18 and 088018, and their phylogenetic relationship within Brassicaceae were described in the present study.

Fresh leaves were sampled from the Jiangsu Academy of Agricultural Sciences, Nanjing, Jiangsu Province (32°2′15″N, 118°52′7″E), China. The voucher specimens were deposited at the herbarium of Key Laboratory of Cotton and Rapeseed, Ministry of Agriculture and Rural Affairs (Feng Chen and 22794238@qq.com) under the accession number of NJ2016Y006 and NJ2016Y003 for NY18 and 088018, respectively. Genomic DNAs were extracted using the modified CTAB method. Paired-end libraries of 450 bp were constructed and sequenced on the NovaSeq system (Illumina, San Diego, CA). SMRTbell DNA libraries (∼10 kb) were prepared using the BluePippin size selection system following the official protocol and sent to Shanghai Biozeron Biotech Co., Ltd for Sequel sequencing (PacBio, Menlo Park, CA). More than 5 Gb Illumina data and 300 Mb PacBio data were obtained for each sample (Table S1). The mitochondrial genomes were hybrid assembled using SPAdes 3.13.0 (Antipov et al. 2016). Additionally, NOVOplasty 4.0 (Dierckxsens et al. 2017) and MITObim 1.9.1 (Hahn et al. 2013) were also applied to fill gaps and verify the assembly. Thereafter, the genomes were annotated using GeSeq (Tillich et al. 2017), coupled with manual correction.

The complete mitochondrial genomes of *B. napus* varieties NY18 and 088018 were double-stranded circular molecules with a length of 221,864 bp and 222,015 bp, respectively. Both genomes encoded 40 protein-coding genes, 21 tRNA genes, and three rRNA genes (Table S2). Among those genes, *ccmFc*, *cox2*, *rps3*, and *rpl2* contained one intron, *nad1* and *nad5* contained two introns, *nad2* and *nad4* contained three introns, and *nad7* contained four introns. The genes *nad1*, *nad2*, and *nad5* were trans-spliced. MUMmer 3.9.2 (Kurtz et al. 2004) was employed to compare the two genomes. Considerable structural variations were existed between the NY18 and 088018 mitochondrial genomes, which were separated into five syntenic regions (each >20 kb) (Figure S1). Although the positions of the syntenic regions between the two mitochondrial genomes were rearranged, the nucleotide sequences of these syntenic regions were highly conserved (sequence identity > 99.99% for each region).

Phylogenetic analysis was performed using 15 complete mitochondrial genomes, and two taxa *Batis maritima* and *Carica papaya* were selected as outgroups (Table S3). Whole genome-wide alignments were achieved by HomBlocks pipeline (Bi et al. 2018). The maximum likelihood (ML) bootstrap analysis with 1000 replicates was performed using RAxML 8.2.12 (Stamatakis 2014). GTR was selected as the best-fit model according to Modeltest3.7 (Posada and Crandall 1998). As shown in Figure 1, phylogenetic tree reveals the topological structure of 15 selected taxa. The three mitochondrial sequences of *B. napus* were closely clustered, forming a single clade which showed a relatively close relationship with the clade formed by *B. rapa*, *B. juncea*, and *B. oleracea*. The data in this study will provide useful information for further evolutionary analysis of *Brassica*.

**Figure 1.**
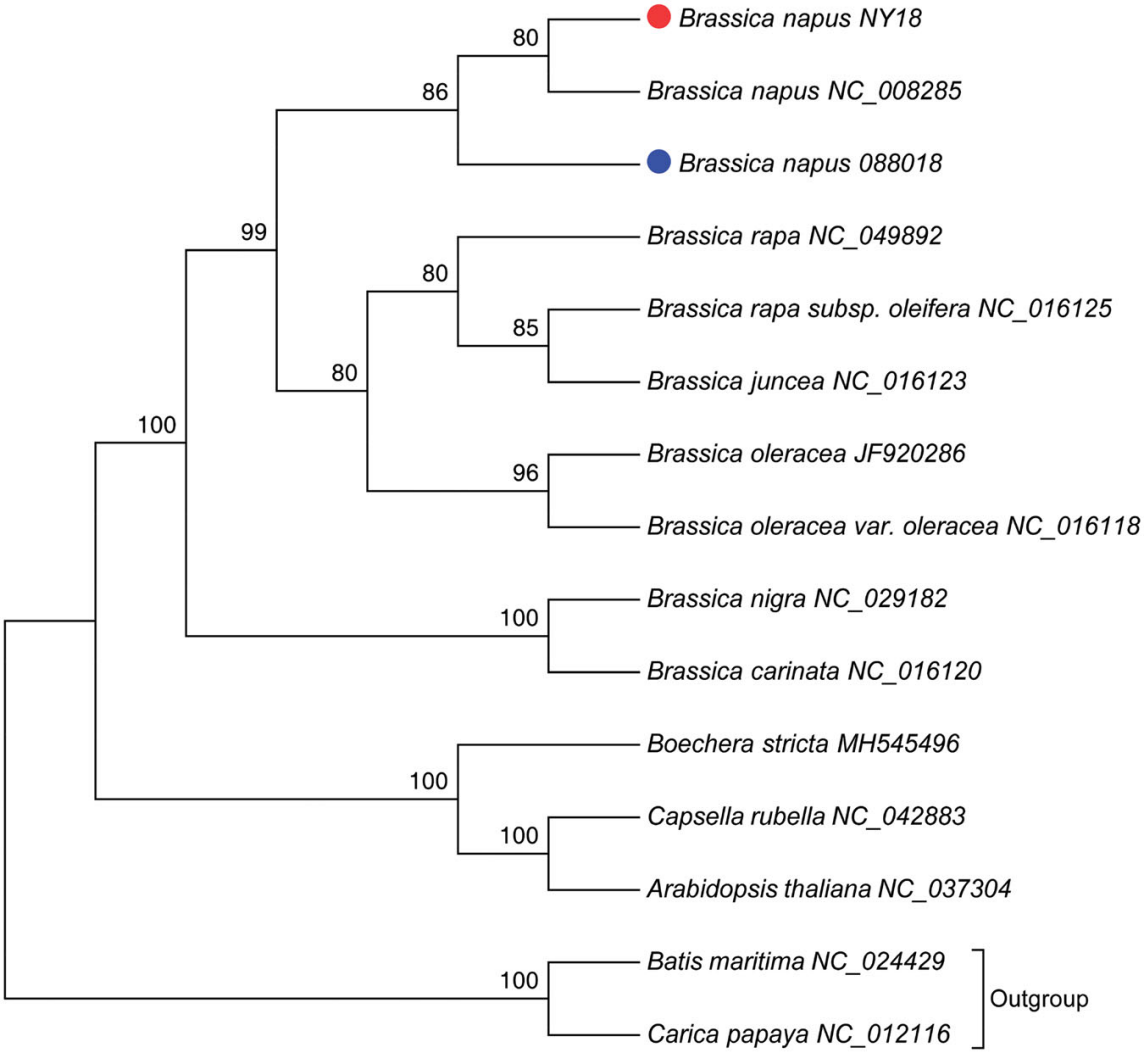
Maximum-likelihood phylogenetic tree based on 15 complete mitochondrial genome sequences.

## Data Availability

The data that support the findings of this study are openly available in GenBank of NCBI at https://www.ncbi.nlm.nih.gov, reference number MW001149 for NY18 and MW348924 for 088018. The associated BioProject accessions of NY18 and 088018 are PRJNA662838 and PRJNA682878, respectively.
